# Expression of *HOTAIR* and *PTGS2* as potential biomarkers in chronic myeloid leukemia patients in Brazil

**DOI:** 10.3389/fonc.2024.1443346

**Published:** 2024-10-10

**Authors:** Ana Paula Kubaski Benevides, Anelis Maria Marin, Denise K. Wosniaki, Rafaela Noga Oliveira, Gabriela Marino Koerich, Bianca Nichele Kusma, Eduardo Cilião Munhoz, Dalila Luciola Zanette, Mateus Nóbrega Aoki

**Affiliations:** ^1^ Laboratory for Applied Science and Technology in Health, Carlos Chagas Institute, Oswaldo Cruz Foundation (Fiocruz), Curitiba, Brazil; ^2^ Hematology and Oncology Clinic, Erasto Gaertner Hospital, Curitiba, Brazil

**Keywords:** CML, biomarkers, lncRNAs, *HOTAIR*, *PTGS2*, RNAseq

## Abstract

Chronic myeloid leukemia (CML) is a clonal myeloproliferative neoplasm in which all the patients has the translocation (*9;22*) that generates de BCR::ABL1 tyrosine kinase. Despite this disease possessing a good biomarker (*BCR::ABL1* transcripts level) for diagnosis and prognosis, many studies has been performed to investigate other molecules, such as the long noncoding RNAs (lncRNAs) and mRNAs, as potential biomarkers with the aim of predicting a change in *BCR::ABL1* levels and as an associated biomarker. A RNAseq was performed comparing 6 CML patients with high *BCR::ABL1* expression with 6 healthy control individuals, comprising the investigation cohort to investigate these molecules. To validate the results obtained by RNAseq, samples of 87 CML patients and 42 healthy controls were used in the validation cohort by RT-qPCR assays. The results showed lower expression of *HOTAIR* and *PTGS2* in CML patients. The *HOTAIR* expression is inversely associated with *BCR::ABL1* expression in imatinib-treated CML patients, and to *PTGS2* showing that CML patients with high *BCR::ABL1* expression showed reduced *PTGS2* expression.

## Introduction

Chronic myeloid leukemia (CML) is a clonal myeloproliferative neoplasm of the hematopoietic system, characterized by the presence of the Philadelphia chromosome (Ph), a fusion chromosome t (*9,22*) (q34;q11) resulting from the reciprocal translocation between chromosomes 9 and 22. This translocation generates a chimeric gene between the *BCR* (breakpoint cluster region protein) and *ABL1* (Abelson murine leukemia viral oncogene homolog 1) genes. This genetic translocation produces a constitutively active tyrosine-kinase, leading to the general imbalance found in CML (1). Notably, CML patients are treated with tyrosine-kinase inhibitors (TKIs), especially with the first-generation TKI imatinib, which shows great efficiency and safety (2). However, about one-third of CML imatinib-treated patients switch to second or third-generation TKIs (dasatinib, nilotinib, ponatinib) due to resistance or toxicity (3).

Long noncoding RNAs (lncRNAs) are noncoding transcripts longer than 200 nucleotides that play crucial regulatory roles in gene expression, translation, genome organization, and cell structure, both in physiological and pathological contexts (4–7). These molecules typically exhibit restricted expression patterns and are often highly cell-specific, except for MALAT1 and NEAT1 (8–10). While many lncRNAs are found in the nucleus, a significant fraction is located in the cytoplasm (11). Nuclear lncRNAs are more abundant but less stable than their cytoplasmic counterparts, and their nuclear instability reflects a fine-tuning regulation of transcriptional programs. In the cytoplasm, lncRNAs mostly sequester miRNAs to regulate their activity and levels, which in turn signaling pathways by regulating miRNA target proteins and mRNAs (12).

Physiologically, lncRNAs display dynamic expression during the differentiation of various cell types, such as muscle, immune, and neural cells, underscoring their role in cellular differentiation (10, 13). Due to their important roles, lncRNAs have gained prominence in scientific and translational research as biomarkers for diagnosis, prognosis, and treatment response or resistance, particularly in the oncology field (14–17). The lncRNA *HOTAIR* has been described in solid tumors, such as breast cancer (18, 19), hepatocellular carcinoma (20, 21), glioma (22, 23) and colon cancer (24, 25), where it plays an oncogenic role related to a worse prognosis and reduced chance of complete remission. A functional mechanism of *HOTAIR* may be represented by its participation and interaction with epigenetic regulators such as the PRC2 complex and the Lysine Demethylase 1 (LSD1) in chromatin remodeling and transcription (26). Furthermore, it was functional associated as regulator of Wnt/β-catenin signaling pathway (27), suppressing *TGF-β1* and *ZEB1* (28) and sponging microRNAs such miR-331-3p (29) and miR-126 (30). One of the first HOTAIR expression role in oncohematology was observed as regulating cell cycle progression during myeloid maturation in human promyelocytic leukemia cells (31) and modulating *c-KIT* through expression sponging miR-193a in acute myeloid leukemia (32). More recently, studies also showed this transcript associated with molecular pathways and process in leukemias (33–35).

The prostaglandin-endoperoxide synthase 2 (PTGS2), also known as cyclooxygenase-2 (COX-2), is an enzyme encoded by the *PTGS2* gene. It plays a role in the conversion of arachidonic acid into prostaglandin H_2_, which is further transformed into five primary prostaglandins (PGD2, PGE2, PGF2α, PGI2, and TXA2) by cell-specific synthases (36). This enzyme is associated with inflammatory diseases, carcinogenesis, angiogenesis, metastasis (37, 38) and apoptosis resistance (39, 40). Its expression pattern is also linked to carcinogenesis (41). *PTGS2* research in oncohematological diseases advanced to a knowledge of its involvement and therapeutical approach (42–44), highlighting it importance as an active player in bone marrow and blood cellular context and homeostasis.

This study aimed to evaluate lncRNAs as potential biomarkers in CML by comparing the transcriptomes of CML patients with high levels of *BCR::ABL1* transcripts to those of healthy donors. The selected transcripts were then validated in a larger cohort of patients, demonstrating the differential expression of several transcripts that may be correlated with CML prognosis. The hypothesis was that mRNA and lncRNA expression in white blood cells from CML patients with *BCR::ABL1* positive/negative expression could represent an *BCR::ABL1* associated biomarker, linking for molecular pathways and preliminary data for further studies.

## Methods

Study cohort — This study was conducted after receiving approval from the Ethics Committee of the Hospital Erasto Gaertner (CAAE 08809419.0.0000.0098) and Hospital do Trabalhador (CAAE 77979417.8.3001.5225). Clinical samples from patients with a confirmed diagnosis of CML and volunteers without a current or previous history of any kind of cancer were recruited from Hospital Erasto Gaertner and Hospital do Trabalhador, in Curitiba, Brazil, from May 2020 to December 2023, following Brazilian guidelines and regulations. The study was described in detail to all participants, who read, discussed, and signed an informed consent form before sample collection. For each patient, 4 mL of peripheral blood was collected in EDTA tubes and processed within 24 hours. CML patients had blood collected several times during treatment and clinical follow-up throughout the project period. The blood was centrifuged to obtain buffy coats and plasma. Personal and clinical data such as age, gender, date of diagnosis, and treatment were also accessed from patients’ clinical records. For RNA sequencing (RNAseq), six samples from CML patients with *BCR::ABL1* expression higher than 10% and treated with imatinib were selected. The control group comprised six healthy volunteers paired for gender and age. For the validation cohort, a total of 87 CML clinical samples from patients under treatment with imatinib were selected and subdivided according to the *BCR::ABL1* expression levels as follows: 30 CML samples with *BCR::ABL1* higher than 2% (*BCR::ABL1* high); 28 CML samples with *BCR::ABL1* lower than 2% (*BCR::ABL1* low), and 29 CML samples with undetectable *BCR::ABL1* expression. The control group included 42 individuals without any current or previous history of any history of cancer, paired for gender and age.

RNA extraction and cDNA synthesis: Total RNA was extracted from the buffy coat using QIAmp RNA Blood Mini Kit (QIAGEN, Hilden, Germany). The extracted RNA was quantified using a NanoDrop™ One spectrophotometer (ThermoFisher, Waltham, MA, USA). cDNA was synthesized from 1µg of total RNA using random primers and SuperScript III (ThermoFisher, Waltham, MA, USA), following the manufacturer’s instructions. The resulting cDNA was then diluted (1:2) and used for validation with RT-qPCR reactions.

*BCR::ABL1* quantification: *BCR::ABL1* expression analysis was performed following an in-house one-step duplex qPCR methodology, as previously described by Marin et al. (2023) (45).

Library construction and sequencing (ribosome RNA depletion): The quantity and purity of the total RNA were assessed using a Bioanalyzer 2100 with an RNA 6000 Nano LabChip Kit (Agilent, CA, USA), and only RNAs with RIN number > 7.0 were used. Approximately 5ug of total RNA was used to deplete ribosomal RNA according to the Ribo-Zero Gold rRNA Removal Kit (Illumina, Cat.MRZG12324, San Diego, USA). After removing ribosomal RNAs, the remaining RNAs were fragmented into short fragments using divalent cations at high temperatures. The cleaved RNA fragments were then reverse transcribed using SuperScriptTM II Reverse Transcriptase (Invitrogen, USA). The cDNA was subsequently used to synthesize U-labeled second-stranded DNAs with *E. coli* DNA polymerase I (NEB, USA), RNase H (NEB, USA), and dUTP Solution (Thermo Fisher, USA). An A-base was added to the blunt ends of each strand to prepare them for ligation to the indexed T-base adapters. Single- or dual-index adapters are ligated to the fragments, and size selection was performed with AMPureXP beads. The ligated products were amplified by PCR, resulting in a final cDNA library with an average insert size of 300 ± 50bp. Finally, 2×150bp paired-end sequencing was performed on an Illumina NovaseqTM 6000, following the vendor’s recommended protocol.

Sequence and filtering of clean reads: A total of million 2 x 150 bp paired-end reads were generated and subsequently filtered using Cutadapt (version: cutadapt-1.9, https://cutadapt.readthedocs.io/en/stable/) with using quality controls parameters. The sequence quality was verified using FastQC, including the Q20, Q30, and GC content of the clean data.

Mapping with reference genome: The reads from all samples were aligned to the human reference genome using HISAT2 (version: hisat2-2.0.4, https://daehwankimlab.github.io/hisat2/) package. HISAT2 allows multiple alignments per read and permits a maximum of two mismatches when mapping the reads to the reference. It also builds a database of potential splice junctions and confirms these by comparing the previously unmapped reads against the database of putative junctions.

Quantification of gene abundance: The mapped reads of each sample were assembled using StringTie (version: stringtie-1.3.4d, http://ccb.jhu.edu/software/stringtie/) with default parameters. Then, all transcriptomes from all samples were merged to reconstruct a comprehensive transcriptome using gffcompare software (version: gffcompare-0.9.8, http://ccb.jhu.edu/software/stringtie/gffcompare.shtml). Once the final transcriptome was generated, StringTie and Ballgown (http://www.bioconductor.org/packages/release/bioc/html/ballgown.html) were used to estimate the expression levels of all transcripts. The expression abundance for mRNAs was quantified by calculating the FPKM (fragment per kilobase of transcript per million mapped reads) value.

Differentially expressed genes (DEGs) analysis: The differential expression analysis was performed using DESeq2 (R package) between two different groups, and by edgeR was used for analysis between two samples. Genes with the parameter of false discovery rate (FDR) below 0.05 and absolute fold change ≥ 2 were considered differentially expressed.

Principal component analysis (PCA): PCA was performed using the princomp function in R (http://www.r-project.org/).

GO enrichment analysis: Gene Ontology (GO) is an international standardized gene functional classification system with three ontologies: molecular function, cellular component, and biological process. All DEGs were mapped to GO terms in the Gene Ontology database (http://www.geneontology.org/). The number of genes associated with each term was calculated, and significantly enriched GO terms in DEGs, compared to the genome background, were identified using a hypergeometric test.

Pathway enrichment analysis (KEGG): Genes usually interact with each other to perform specific biological functions. Pathway-based analysis helps to further understand the biological functions of genes. KEGG is a major public pathway-related database used for this purpose.

Gene set enrichment analysis (GSEA): We performed gene set enrichment analysis using the GSEA (v4.1.0) software and MSigDB database to identify whether a set of genes in specific GO terms and KEGG pathways with significant differences in two groups. Briefly, we input the gene expression matrix and rank genes by the Signal2Noise normalization method. Enrichment scores and p-values were calculated using default parameters. Parameters meeting the conditions of |NES|>1, NOM p-val<0.05, and FDR q-val<0.25 were considered significantly different between the two groups.

RT-qPCR: cDNAs were used as templates in RT-qPCR using TaqMan™ specific assays (ThermoFisher Scientific, Inc., MA, USA) and TaqPath™ Pro Amp™ Master Mix (ThermoFisher, Waltham, MA, USA). B-Actin and GAPDH were selected as housekeeping genes using TaqMan assays with the same amplification protocol. All reactions were carried out in a QuantStudio™ 5 Flex (Applied Biosystems, Foster City, CA, USA), with the following thermal cycling conditions: 50°C for 2 minutes, 95°C for 10 minutes, followed by 40 cycles of 95°C for 15 seconds and 60°C for 1 minute.

Housekeeping selection: the initial analysis excluded all outlier samples using the Outlier calculator from GraphPad Prism (alpha value = 0.05), based on the Ct values of each housekeeping gene from all 129 clinical samples. Subsequently, the best housekeeping gene was selected using BestKeeper software (Pfaffl et al., 2004), using the Ct values for *GAPDH* and *Beta-Actin* from samples not eliminated in the previous step.

Statistical analysis: For gender data chi-square test was used, while for age was used t-student. For qRT-PCR data the relative expression was calculated using the 2-ΔΔCt method, with one healthy control (HC) sample defined as the calibrator, and t-student test was used. All statistical analyses were used as significant p value > 0,05.

## Results

### Study cohort description

The cohort description is described in **Tables 1**, **2**, where the first one shows the characterization of CML patients subjected to RNASeq, indicating age, gender, BCR::ABL1 percentage, and clinical data for blood cells count and creatinine.

**Table 1:** Demographic and clinical data for 6 CML patients subjected to RNASeq, indicating BCR::ABL1 expression, blood cell count and creatinine quantification.

ID	Age ± SD	Gender	BCR::ABL1%	Total leukocytes^*^	Lymphocytes^*^	Neutrophils^*^	Basophils^*^	Platelets^*^	Creatinine^**^
CML Ao10	50	M	18,72	7.110	1.920	4.479	0	264.000	1,1
CML Ao13	49	F	20,52	5.230	2.249	2.563	52	174.000	0,6
CML Ao14	38	M	34,13	4.900	2.352	2.205	0	41.000	1,1
CML Ao15	63	M	12,62	2.820	1.523	1.128	0	27.000	0,8
CML Ao16	60	M	13,05	4.470	626	3.572	0	110.000	1,2
CML Ao17	69	F	37,35	4.190	712	2.682	84	387.000	1,27

M, Male; F, Female; *Cells/mm^3^, ^**^mg/dl.

**Table 2:** Study cohort characterization, describing sample number, mean age, and gender for CML and control groups.

Group	Samples	Mean age ± SD (years)	Gender		BCR::ABL1 expression range	White blood cells per mm3 (mean±StDev)
			Male	Female		
*BCR::ABL1* high	30	54.03 ± 15.53	63.33%	36.67%	82,84%-34,1%	51,726±115,326
*BCR::ABL1* low	28	50.68 ± 12.76	64.29%	35.71%	1%-0,008%	6,927±2,518
undetectable *BCR::ABL1*	29	62.36 ± 11.82	44.83%	55.17%	ND	6,454±1,801
Healthy control	42	54.65 ± 12.28	40.48%	59.52%	ND	NA

ND, Not detected; NA, Not available.

The **Table 2** shows the characterization of all CML and healthy controls study cohort. There were no significant differences in age or gender between the groups. The mean *BCR::ABL1* levels for the *BCR::ABL1* high and *BCR::ABL1* low groups were 82.84% and 0.145%, respectively. The white blood cells and blast count varies especially in high *BCR::ABL1* high expression patients, where from thirty patients eight showed high white blood cells count (ranging from 39,300 to 471,180/mm^3^). For *BCR::ABL1* low and absent expression, white blood cells count were in reference range. Just 4 patients of *BCR::ABL1* high expression group had blast detection with percentage ranging from 3% to 5%.

### RNA sequencing

**Figure 1:**
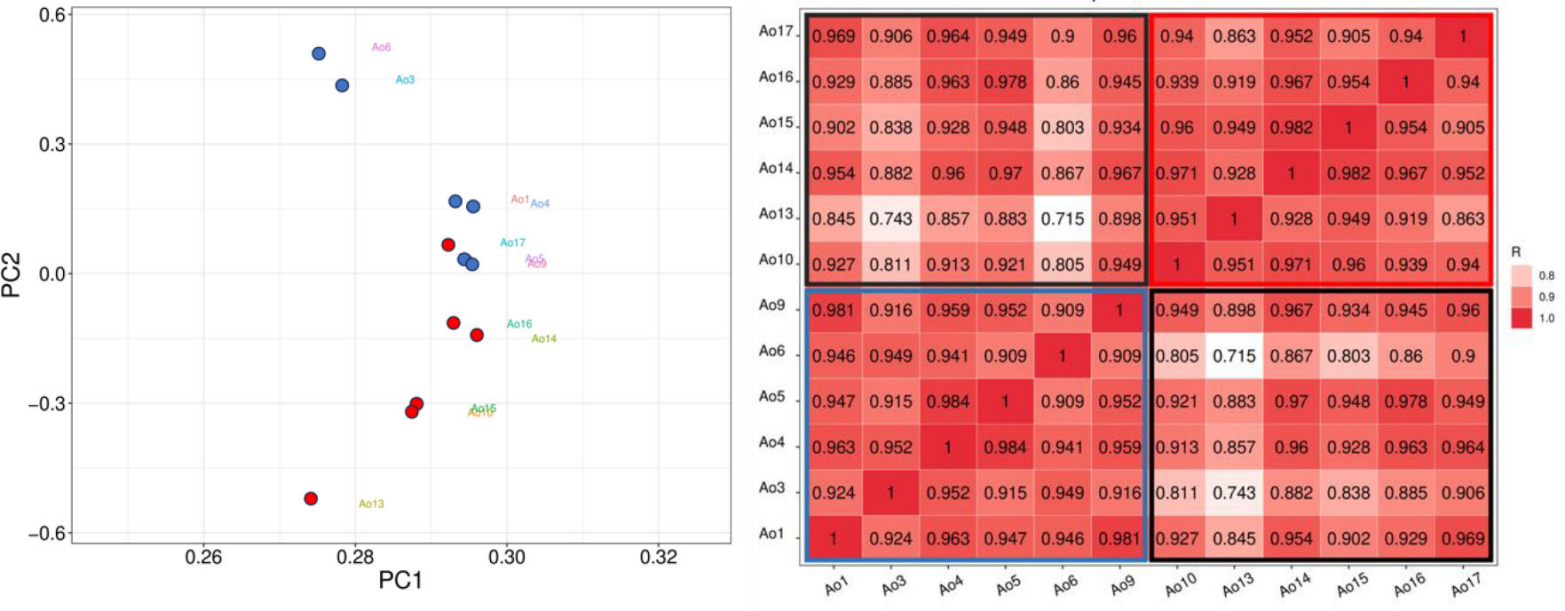
Correlation analyses between CML and HC groups. (Left) PCA analysis demonstrates a separation between CML samples (red dots) and healthy subjects (blue dots), except for one CML sample (Ao17), which clustered with the HC samples. (Right) Person correlation between samples, showing CML sample correlations in upper right (red box), and HC sample correlations in lower left (blue box), and CML *vs* HC sample correlations in the box delimited by black line.

To clarify, HC samples were labeled as Ao1, Ao3, Ao4, Ao5, Ao6, and Ao9, while CML samples were labeled as Ao10, Ao13, Ao14, Ao15, Ao16 and Ao17. The principal component analysis (PCA) showed that only one CML sample (Ao17) clustered with the HC samples (**Figure 1A**). As expected, the Pearson correlation between samples indicated a higher correlation with the CML group and HC group separately, and a lower correlation when comparing CML to HC (**Figure 1B**).

**Figure 2:**
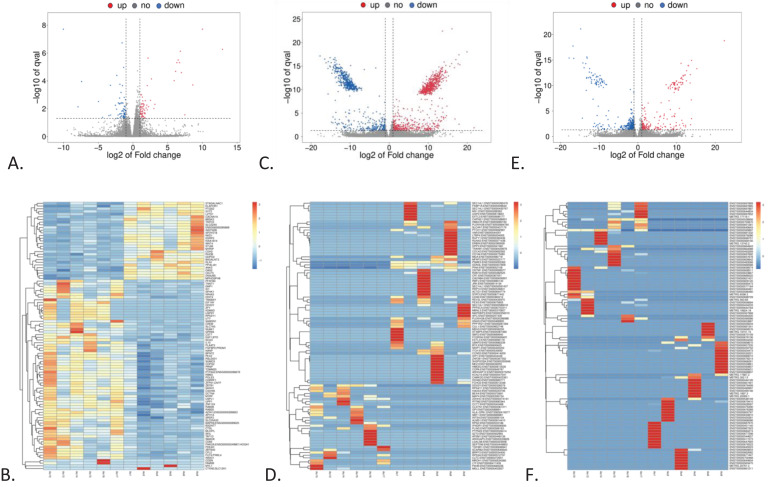
RNAseq general data from CML *versus* healthy control groups relative to genes, transcripts, and lncRNA. **(A)** Volcano plot showing differentially expressed genes in CML *vs* HCs (HC). **(B)** Heatmap demonstrating individual expression of the most differentially expressed genes. **(C)** Volcano plot showing differentially expressed transcripts in CML *vs* HC. **(D)** Heatmap demonstrating individual expression of the most differentially expressed transcripts in CML *vs* HC. **(E)** Volcano plot showing differentially expressed lncRNAs in CML *vs* HC. **(F)** Heatmap demonstrating individual expression of the most differentially expressed lncRNAs in CML versus HC.

The RNAseq analysis identified 133 genes with significantly different expression between CML and the HC group. Of these, 67 genes were upregulated, and 66 genes were downregulated in CML compared to healthy subjects (**Figures 2A, B**). When the analysis was extended to transcripts, 1,621 transcripts showed differential expression between CML and HC groups, from with 953 upregulated and 668 downregulated in CML compared to HC (**Figures 2C, D**). In a more restricted analysis, 583 lncRNAs were differentially expressed, with 224 upregulated and 359 downregulated in CML compared to healthy subjects (**Figures 2E, F**).

**Figure 3:**
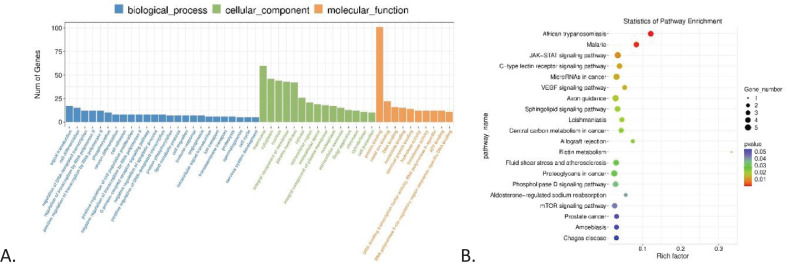
GO and KEGG enrichment analysis. **(A)** GO enrichment analysis of differentially expressed genes in CML, showing the number of genes correlated with biological process, cellular component, and molecular function, highlighting signal transduction, membrane, and protein binding, respectively. **(B)** KEGG pathway enrichment analysis of differentially expressed genes in CML, highlighting JAK-STAT signaling pathway and microRNAs in cancer.

GO enrichment and KEGG pathways analyses for mRNA are shown in **Figure 3**, highlighting an enrichment of protein binding in the Molecular Function component (3A) and the *JAK-STAT* signaling pathway (3B).

**Figure 4:**
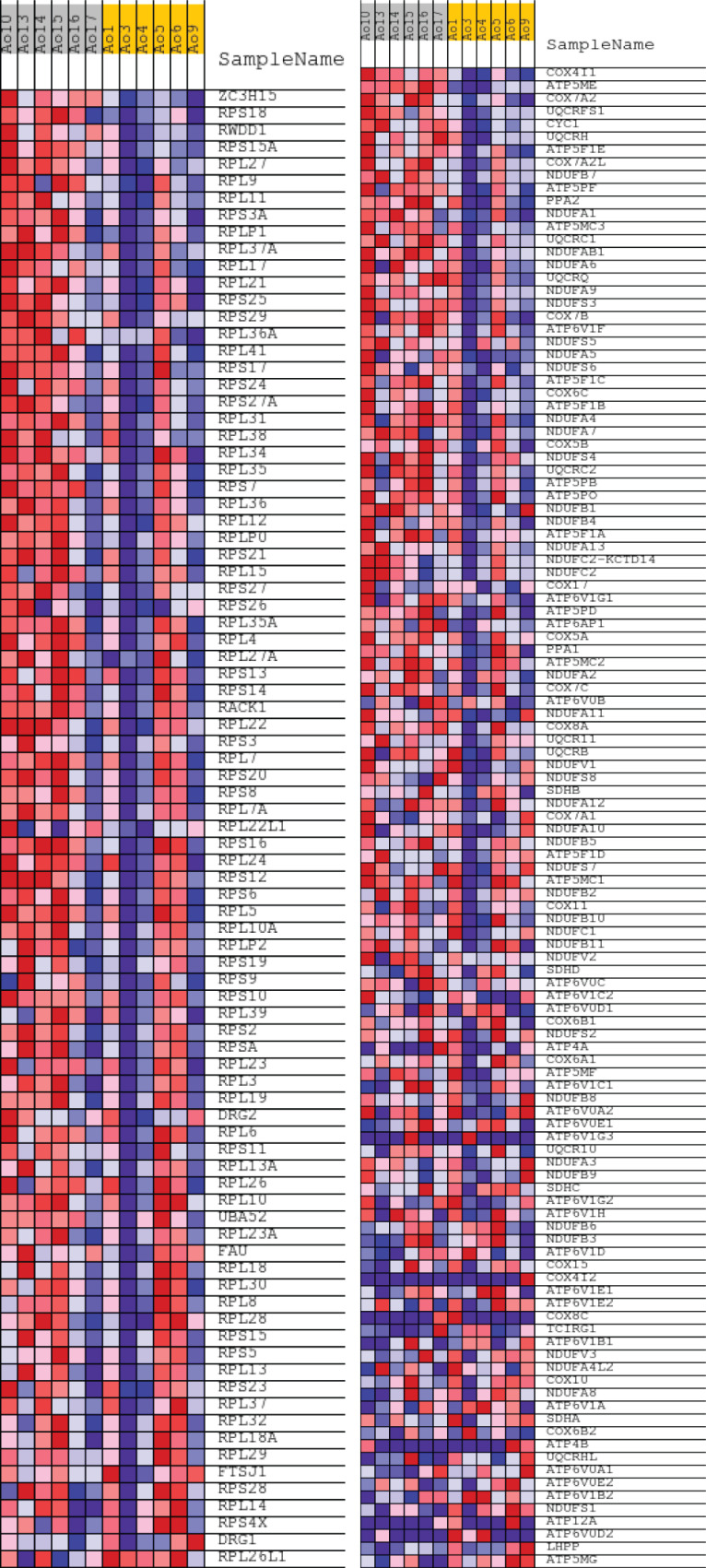
Gene Set Enrichment Analysis (GSEA) for RNASeq of CML and healthy controls exemplifying GO and KEGG pathways for cytoplasmatic translation (left) and Oxidative Phosphorylation (right), respectively.

For GSEA data, it was analyzed compared GO and KEGG showed 76 and 37 statistical different functions/pathways between CML and healthy controls, respectively, exemplifying “cytoplasmatic translation” in GO and “Oxidative Phosphorilation” in KEGG, with size of 87 and 117, respectively, demonstrated in **Figure 4**.

### Validation by RT-qPCR

A critical analysis of differentially expressed mRNA and lncRNA in CML samples was performed with the following criteria: 1) only genes with fold changes higher than 1.2 or lower than 0.5 were considered; 2) Sample positivity percentage (SPP) was used to indicate the number of samples with detectable levels of a given gene/lncRNA within the two groups, CML or HC. A 100% SPP value indicates a gene/lncRNA expressed in all samples from that group, while 0.00% refers to undetectable levels of mRNA/lncRNA in any of the samples in the group. **Table 3** describes this critical analysis of the mRNAs and lncRNAs selected from RNAseq for the validation cohort. Additionally, three lncRNAs (*MALAT, HOTAIR*, and *Ddx3y*) that did not show a statistical difference in RNAseq results but are correlated with oncohematological diseases (though not yet described in CML) according to scientific literature were included.

**Table 3:** mRNAs and lncRNAs selected from RNA sequencing for the validation cohort by RT-qPCR.

Transcript	Name	CML		Healthy controls		Fold-change	Regulation in RNA Seq
		Sample Positivity % (SPP)	FPKM Mean (SD)	Sample Positivity % (SPP)	FPKM Mean (SD)		
mRNA	*PTGS2*	100.00%	24.26 (14.90)	100.00%	104.92 (54.42)	-2.11	Down
	*RHOB*	100.00%	29.71 (10.48)	100.00%	70.10 (17.48)	-1.24	Down
	*CD83*	100.00%	19.18 (13.41)	100.00%	5.57 (1.55)	1.78	Up
	*RPS4Y1*	100.00%	14.37 (10.90)	83.33%	0.13 (0.10)	6.83	Up
lncRNA	*ARHGEF1*	83.33%	2.25 (1.39)	0.00%	ND	NA	Up
	*SSR2*	33.33%	3.92 (6.43)	0.00%	ND	NA	Up
	*ADGRE5*	100.00%	5.65 (0.85)	100.00%	16.13 (9.79)	0.35	Down

SPP, Sample Positivity Percentage.

FPKM, Fragments Per Kilobase Million.

Selected transcripts demonstrating the percentage of samples expressing the transcript, FPKM mean and standard deviation (SD), fold change and regulation in CML patients in correlation with HC. ND, not detected; NA, not available.

**Figure 5:**
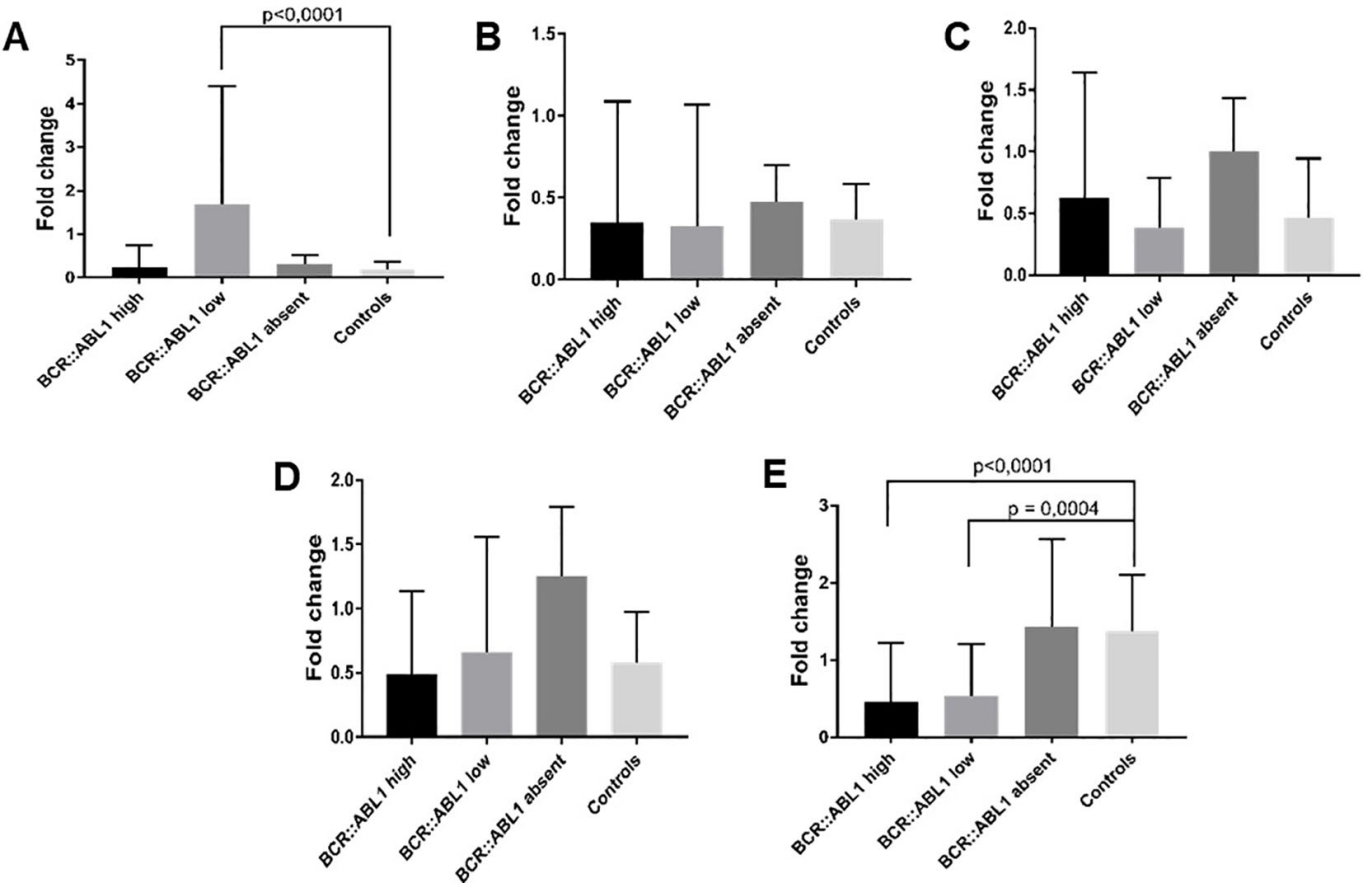
lncRNA expression validation by RT-qPCR. **(A)** *MALAT*, **(B)** *Adgre5*, **(C)** *Arghef1*, **(D)** Ssr2 and **(E)** *HOTAIR* in *BCR::ABL1* high, low, and absent expression and healthy controls. The results indicate that *MALAT* was significantly overexpressed in *BCR::ABL1* low expression samples compared to healthy controls, while *HOTAIR* was down-expressed in both *BCR::ABL1* high and low expression samples compared to controls.

From the 129 clinical samples, one sample from the *BCR::ABL1* low group and four samples from the HC group were removed due to outlier criteria and/or housekeeping Ct values higher than 32, indicating poor nucleic acid quality. Following this, housekeeping gene selection with BestKeeper indicated that *GAPDH* was the most stable option; hence, it was used for normalization. For MALAT it was observed a significant higher expression in *BCR::ABL1* lower expression in comparison to healthy controls (**Figure 5A**), while for *Adgre5*, *Arghef1* and Ssr2 showed no different expression (**Figures 5B–D**). However as depicted in **Figure 5E**, the lncRNA validation showed that *HOTAIR* expression was significantly lower in the CML *BCR::ABL1* high and *BCR::ABL1* low groups compared to the *BCR::ABL1* absent group and HC groups.

**Figure 6:**
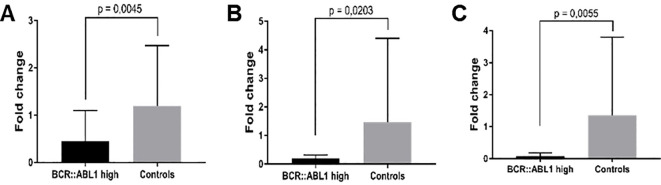
mRNA expression validation by RT-qPCR. **(A)** *PTGS2*, **(B)** *Rhob*, and **(C)** *Cd83* in *BCR::ABL1* high expression patients and HC subjects.

Only samples from *BCR::ABL1* high and HC groups were used for mRNA validation. **Figure 6** shows the significantly lower expression of *PTGS2* (6A), *Rhob* (6B), and *Cd83* (6C) in CML patients compared to HC subjects.

Following this, a critical analysis of *PTGS2* and *HOTAIR* expression in *BCR::ABL1* high-expression patients was performed by subdividing the samples according to their fold change, considering a fold change higher than 1.5 and lower than 0.1. Regarding no finding about *HOTAIR* expression, an interesting data was found in *PTGS2*, where **Table 4** lists the 13 patients included in this subgroup; five presented a higher fold change while eight showed a lower fold change. Interestingly, all patients with PTGS2 fold change ≥1.5 experienced successful imatinib treatment, with no need to switch to another class of TKI. Conversely, six (75%) of the eight patients with a *PTGS2* fold change ≤0.1 had unsatisfactory results with imatinib and therefore had to switch their treatment to the second-line TKI, dasatinib. The follow-up for these cohorts ranged from 1,066 to 532 days, a sufficient period to demonstrate whether the patient responded to imatinib.

**Table 4:** *PTGS2* fold change in CML patients.

*PTGS2* (relative expression)	TKI		Follow-up (days)
1.9643	I		1,066
2.3022	I		1,008
1.6189	I		874
4.2752	I	TFR	889
2.6440	I		890
0.0742	I	D	763
0.0730	I	D	749
0.0219	I	D	637
0.0218	I		742
0.0789	I	D	532
0.0352	I	D	667
0.0938	I		640
0.0745	I	D	532

The patient’s treatment during the follow-up, indicating if the TKI was changed from imatinib to dasatinib. I, imatinib; D, dasatinib; TFR, treatment-free remission.

## Discussion

CML was the first disease to have a targeted therapy, with the development of imatinib, a TKI that specifically targets the BCR::ABL1 protein in leukemia cells (1). However, about 10-15% of CML patients develop resistance to imatinib, which led to the development of other generations of TKIs, such as dasatinib and Nilotinib, including clonal evolution, mutations in the *BCR::ABL1* kinase domain, and activation of *BCR::ABL1* independent pathways (46–48).

We describe here a comparative transcriptome analysis of CML patients and HC subjects, highlighting the lower expression of *HOTAIR* and *PTGS2* in CML patients, which may be related to disease status and imatinib treatment. Despite the pivotal role of the Ph chromosome in CML, some studies have explored the transcriptome of CML patients in search of additional lncRNA and mRNA biomarkers. Giustacchini et al. (2017) conducted a single-cell transcriptomic analysis of CML patients and found an expression signature for normal hematopoietic stem cells (HSCs), BCR::ABL1^+^ and BCR::ABL1^-^, including transcripts previously implicated in CML pathogenesis. When the authors analyzed the top 245 differentially expressed genes, BCR::ABL1^+^ cells clustered separately from BCR::ABL1^-^ cells, indicating a specific, consistent transcriptomic profile across different CML patients (49). BCR-ABL^+^ stem cells (SCs) at diagnosis did not cluster according to response category, with most BCR::ABL^-^ SCs from poor-responder patients contained within the poor-responder cluster of genes. They also identified a subpopulation of highly quiescent BCR::ABL^+^ SCs, already present at diagnosis and markedly selected during treatment in patients who failed to achieve a deep molecular response (49). Youn et al. (2021) identified 398 differentially expressed genes (258 upregulated and 140 downregulated) in pediatric CML CD34+ cells compared to adult ones. Rho pathways were most significantly downregulated in pediatric CML patients, highlighting VAV2 and ARHGAP27 (50).

*PTGS2* codes for an enzyme that belongs to a family responsible for catalyzing the synthesis of prostaglandins from arachidonic acid, which is a stress-inducible protein typically expressed at low levels under normal physiological conditions (51). Its biological function and activation are directly induced by pro-inflammatory cytokines and growth factors correlated with activated intracellular inflammation-related pathways (52). Several reports indicate that *PTGS2* expression levels are directly related to the carcinogenesis process, particularly due to the inflammatory and, consequently, immunological context. In innate immunity, natural killer cells are inhibited from exerting cytotoxic effects, migrating and secreting interferon γ by PGE2, the main enzymatic product of PTGS2 activity (53, 54). Göbel et al. (2014) showed that *PTGS2* overexpression can initiate and promote carcinogenesis by inhibiting the proliferation of B and T lymphocytes, particularly natural killer T cells, thereby acting as an immunosuppressor (55). PGE2 directly inhibits the proliferation and effector functions of CD4+ and CD8+ T cells and promotes their differentiation in regulatory T cells (Tregs), contributing to the immune response associated with melanoma (56). Several reports have linked the immune system, cancer, and *PTGS2*, demonstrating that its expression induces Tregs, and these cells support cancer-mediated immune suppression (57). PGE2 is directly involved in the complex tumor inflammatory microenvironment, inhibiting inflammatory chemokines CCL3 and CCL4, preventing the accumulation of activated immune cells. It also reduces IL-10 secretion and shifts the microenvironment in favor of Th1 immune response (58).

There are only a few reports of *PTGS2* in the oncohematological context, with inconclusive results regarding its role in leukemias. A recent study on Mexican adults with acute lymphoblastic leukemia showed that *PTGS2* is highly downregulated compared to controls using transcriptomic microarray analysis (59). In a recent lymphoma report, Qi et al. (2023) demonstrated a consistent correlation between *PTGS2* upregulation and chromosome 17p deletions in human B-cell lymphomas, linking arachidonate metabolism as a new susceptibility factor for lymphoma (60). Regarding CML, the role of *PTGS2* remains unclear primarily due to a lack of clinical studies. However, Giles et al. (2002) showed higher PTGS2 protein levels in the bone marrow of 149 chronic-phase CML patients compared to normal controls, and higher PTGS2 levels were significantly associated with shorter survival. Most PTGS2 studies in CML are *in vitro*, focusing on pharmacologically suppressing *PTGS2* activity (60). Celecoxib, a specific *PTGS2* inhibitor, has been shown to reduce the growth of both K562 and primary CML cells and induce apoptosis in a dose-dependent manner. This effect is accompanied by the downregulation of cyclin D1, cyclin E, and p-Rb expression, the upregulation of *P16(INK4a)* and *P27KIP* expression, and a G1-S phase arrest of the cell cycle (61). Another study using PTGS2-inhibitors showed similar results of reducing growth and inducing apoptosis in a K562 cell line, including indomethacin (62), DuP-697 (63) and nabumetone (64). Together, these results indicate that the *in vitro* pharmacological suppression of *PTGS2* may positively affect CML treatment. However, our study presents a controversial result, showing that CML patients with high *BCR::ABL1* expression showed reduced *PTGS2* expression.

The lncRNAs are non-protein coding RNAs enrolled in several biological processes, including the regulation of gene expression under both normal physiological and disease conditions (65). Recent results have shown that the intracellular location of lncRNAs is crucial; those located in the nucleus can regulate chromatin and transcription, while the ones in the cytoplasm are involved in mRNA stability and translation regulation (66). LncRNAs have been implicated in the initiation and progression of leukemia (67), *NEAT1* (nuclear paraspeckle assembly transcript 1) is correlated with the poor progression in CML (68) and with multidrug resistance in pediatric acute lymphocytic leukemia (69). Other lncRNAs, such as *MALAT* (70), *GAS5* (71), *ANRIL* (72), *TUG* (73) and *PANDAR* (74) have been shown to be downregulated in lymphoid and myeloid leukemias, underscoring their significance in hematological malignancies.

We found that lncRNA *HOTAIR* expression is inversely associated with *BCR::ABL1* expression in imatinib-treated CML patients, as those with absent *BCR::ABL1* showed *HOTAIR* expression levels similar to control subjects. Its lower expression may represent a biomarker associated with *BCR::ABL1* expression, even in early disease or in response to TKI treatment. In oncohematology, *HOTAIR* high expression correlates with the poor prognosis in acute myeloid leukemia, suggesting a relationship with exacerbated proliferation of SCs, a higher number of blasts, and lower disease-free and overall survival (75) Information on *HOTAIR* expression in CML is limited; still, Wang et al. (2017) showed that *HOTAIR* was significantly upregulated in multidrug resistance protein 1 positive patients and in the K562-imatinib-resistant cells. Knockdown of *HOTAIR* in the K562-imatinib-resistance cells resulted in higher sensitivity to imatinib treatment and attenuation of the PI3K/Akt pathway, suggesting that *HOTAIR* plays a crucial role in acquired resistance to imatinib (76). Two reports showed *HOTAIR* expression in bone marrow of Chinese CML patients, divided into chronic phase, accelerated phase and blast crisis. Contrary to our finding, both reports showed a significant higher expression of HOTAIR in CML patients in comparison to healthy controls, however both also demonstrated that accelerated phase and blast crisis samples showed HOTAIR expression significantly higher than chronic phase (77, 78). It was also demonstrated that *HOTAIR* had higher expression in K562 and KCL-22 cells compared to bone marrow mononuclear cells, accompanied by lower expression of PTEN, and functional results indicated that *HOTAIR* downregulation reduces the proliferation, colony formation, and cell cycle progression while increasing the apoptosis rate of CML cells (78).

So here we described the transcriptomic profile of *BCR::ABL1* Brazilian CML patients and demonstrated differential expression of mRNA and lncRNA compared to healthy controls subjects. Selected transcripts were validated by RT-qPCR in a separate cohort, confirming the downregulation of *HOTAIR* and *PTGS2* in *BCR::ABL1* high-expression Brazilian CML patients. Our results suggest a relationship between imatinib response and downregulation of *PTGS2* and *HOTAIR*, as CML patients with higher expression of these transcripts successfully responded to imatinib. Despite the patient stratification according to *BCR::ABL1* expression levels, a weakness of this study is was the use of white blood cells, not analyze specific cell subsets, such as CD34+ cells, and instead examined a heterogeneous population of peripheral blood leukocytes. This results are limited and described to this scenario, however comprises data that *HOTAIR* and *PTGS2* expression is not consolidated in CML studies, variating according study design and intended goals. However, as a strength considering the results were validated in a larger cohort with extensive clinical data and long follow-up, supporting the value of our findings. It was observed that HOTAIR and PTGS2 may represent an important role in CML progression, as biomarker of TKI efficiency and resistance, however more studies are required, especially for basic cellular and molecular contexts. Additionally, our group is following and generating data on specific cell type and follow-up recruitments in CML patient, aiming describing more specific and detailed findings.

## Data Availability

The raw data supporting the conclusions of this article will be made available by the authors, without undue reservation.
